# Life Cycle Assessment of Polyphenolic Extracts Derived from Pine By-Products

**DOI:** 10.3390/ma18051000

**Published:** 2025-02-24

**Authors:** Grau Baquero, Sílvia Sorolla, Concepció Casas, Anna Bacardit

**Affiliations:** A3 Leather Innovation Center, Escola Politècnica Superior, Departament d’Informàtica i Enginyeria Industrial, Universitat de Lleida (UdL), 25003 Lleida, Spain; grau.baquero@udl.cat (G.B.); silvia.sorolla@udl.cat (S.S.); concepcio.casas@udl.cat (C.C.)

**Keywords:** polyphenolic extracts, pine by-products, life cycle assessment, atomization, freeze-drying

## Abstract

Forestry and wood-processing by-products, such as pine bark, offer promising opportunities for sustainable resource utilization within a circular economy. This study aimed to assess the environmental impact of an aqueous extraction process for polyphenolic compounds from various pine residues, including bark, cones, and pruning, using life cycle assessment (LCA). The analysis revealed that ground and sieved pine bark powder had the lowest environmental impact, attributed to its simpler extraction process without chemical modifications and reduced energy consumption compared to other pine-derived products. Electricity and natural gas were identified as the primary drivers of environmental impacts across all categories. Sensitivity analyses demonstrated that increasing the tannin concentration in pine-derived products and integrating renewable energy sources could further improve environmental performance. These findings highlight the potential of utilizing underutilized pine residues as sustainable feedstock for producing valuable polyphenolic extracts with a relatively low environmental footprint. The insights gained from this LCA study provide a comprehensive foundation for advancing sustainable extraction technologies. They emphasize the critical role of energy efficiency, tannin concentration, and renewable energy integration in minimizing environmental impacts. Furthermore, these findings offer actionable guidance for optimizing resource recovery from forestry by-products, enhancing their viability as eco-friendly alternatives to conventional tannin sources.

## 1. Introduction

The transition toward sustainable development and circular economic models has intensified the focus on valorizing renewable resources, particularly through bio-based chemical production. Among these resources, biomass derived from forestry and wood-processing industries is gaining recognition as valuable feedstock with considerable economic potential. This biomass can be converted into high-value products and energy, thereby addressing the global demand for sustainable resource utilization [1].

Forestry and wood-processing by-products, such as wood bark, align with circular economy principles as promising feedstock for recycling and closing product life cycles. Globally, the forestry industry generates approximately 600 million cubic meters of by-products annually, including bark, sawdust, and wood chips, with bark alone constituting around 10–15% of a tree’s total volume [2]. Simultaneously, the need for sustainable industrial processes has established renewable biomass as a critical foundation for developing bio-based chemicals.

Polyphenolic compounds, such as tannins, are notable among bio-based chemicals due to their versatile applications in sectors including pharmaceuticals, food preservation, cosmetics, and leather processing [3,4,5]. Traditionally extracted from sources like quebracho and mimosa, tannins possess functional properties such as antioxidant and antimicrobial activities. However, conventional extraction methods often incur high energy consumption and significant environmental impacts, underscoring the necessity for more sustainable approaches.

Conventional extraction methods typically involve the use of high-temperature water or organic solvents to isolate tannins from plant materials. These processes are energy-intensive, with temperatures often exceeding 100 °C and prolonged extraction times that can range from several hours to days. For instance, aqueous extraction methods may consume up to 10 MJ/kg of raw material, while organic-solvent-based methods can require even higher energy inputs due to additional steps like solvent recovery and distillation.

The valorization of forestry by-products, particularly those derived from pine, has emerged as a promising strategy due to their rich polyphenol content and alignment with sustainable development goals. Pine residues, including bark, needles, and cones, are widely available and underutilized by-products of the forestry industry. These residues are abundant in polyphenolic compounds such as flavonoids, tannins, and phenolic acids, which exhibit bioactivities including antioxidant, anti-inflammatory, and antimicrobial properties [6,7,8].

Recent advancements in extraction techniques have demonstrated the potential to efficiently recover these valuable compounds from pine residues. While traditional solid–liquid extraction using organic solvents remains prevalent, it is often associated with high solvent consumption and energy demand. To mitigate these limitations, greener and more efficient methods such as ultrasound-assisted extraction (UAE) [9,10,11,12], microwave-assisted extraction (MAE) [13,14,15,16,17,18], and supercritical fluid extraction (SFE) [19,20] are increasingly being employed. These techniques reduce energy consumption, minimize the use of harmful solvents, and often yield higher concentrations of polyphenolic compounds [21,22].

Among pine-derived by-products, bark is particularly noteworthy for its high content of condensed tannins and other phenolic compounds. Proanthocyanidins extracted from pine bark exhibit strong antioxidant and radical-scavenging activities, making them attractive for applications in nutraceuticals, food packaging, and personal care products [23,24]. Additionally, enzymatic hydrolysis and other biotechnological innovations have been explored to enhance extraction efficiency and improve the functional properties of these compounds [25].

Beyond bark, pine needles and cones also possess unique polyphenolic profiles with potential industrial applications. Pine needles are rich in phenolic acids and lignans with notable bioactivity, while cones provide tannin-rich extracts that are applicable in leather tanning and wood adhesive formulations [26,27,28].

The extraction and processing of tannins and other polyphenolic compounds, however, are often energy-intensive and pose environmental challenges. Assessing the environmental impacts of these processes through life cycle assessment (LCA) is critical for guiding the development of sustainable technologies [29,30]. For instance, Carlqvist et al. [1] identified ethanol consumption and electricity usage as primary contributors to the environmental burden of extraction methods such as hot water extraction (HWE), UAE, and SFE. Similarly, Barjoveanu et al. [31] highlighted the importance of solvent recovery and energy efficiency in mitigating the ecological footprint of polyphenol extractions.

In previous studies, the potential application of polyphenols derived from Pinus halepensis bark, sourced from Mediterranean forests in Spain, was investigated. The bark was milled into a fine powder, with particle sizes typically around 50 µm. Without undergoing any chemical treatment, the pine bark powder was found to have a tannin content of 26.2%. Subsequently, a life cycle assessment (LCA) was conducted to compare the environmental impact of the pine bark powder and atomized mimosa extract.

Specifically, the LCA evaluated two aspects: (i) the production of pine bark powder tannins and atomized mimosa extract as raw materials, and (ii) the tanning process of leather using pine bark tannins versus mimosa extract. Utilizing pine bark powder for tanning resulted in an 83% reduction in the “climate change” impact category. However, when the pine bark tannin was applied in leather tanning, the process exhibited increased environmental impacts across all evaluated categories compared to mimosa extract. This was attributed to the high insoluble matter content of the pine bark tannin.

To address this limitation, the current investigation builds upon previous work [32,33] by conducting a comprehensive LCA of aqueous tannin extraction from pine by-products, with particular attention given to the scalability challenges associated with freeze-dried and atomized products. By identifying environmental hotspots and analyzing scalability constraints, this study seeks to optimize resource utilization and minimize ecological impacts in tannin production systems.

## 2. Materials and Methods

Following the hypothesis that the environmental impact of polyphenolic extraction from pine by-products can be significantly reduced by optimizing tannin concentration and transitioning to renewable energy sources for the extraction and concentration processes, this study examined the production of polyphenolic extracts from three forestry by-products of pine trees: bark, cones, and pruning. The extracts were obtained through aqueous extraction using an autoclave and subsequently concentrated through atomization or freeze-drying to enhance their tannin yield. Comparative data for commercial tannins—mimosa, quebracho, and tara—were included to benchmark environmental impacts. Additionally, previous results of ground pine bark powder sieved to 2 mm were compared [33].

The atomization of vegetable extracts is a process that transforms liquid extracts into fine powders by breaking the liquid into small droplets and removing the water content through evaporation. This process is primarily used in the production of powders from liquid plant extracts, offering advantages such as improved shelf stability, ease of handling, and preservation of bioactive compounds. The atomization process involves several key steps:

‑ Liquid Dispersion: The vegetable extract, which is typically a concentrated aqueous solution containing bioactive compounds like polyphenols or essential oils, is first prepared. It is then fed into an atomizer, a device that uses mechanical or pneumatic energy to disperse the liquid into a fine mist of tiny droplets.
‑ Droplet Formation: In the atomizer, the liquid is forced through a nozzle or sprayed by high-speed rotation, breaking it up into fine droplets. These droplets are small enough to have a high surface area, which is crucial for rapid drying.
‑ Drying: Once atomized, the droplets are introduced into a hot air stream within a drying chamber. The heat causes the moisture within the droplets to evaporate quickly, leaving behind solid particles. The temperature and airflow are carefully controlled to avoid the thermal degradation of active compounds in the vegetable extract.
‑ Collection: The dried particles, now in the form of a fine powder, are collected at the bottom of the drying chamber or separated through a cyclone system. The powder is then cooled to prevent aggregation or caking and is packaged for storage or further use.

In contrast, lyophilization, also known as freeze-drying, is a dehydration process used to preserve vegetable extracts by removing water while maintaining the structural integrity and bioactive compounds of the extract. This process is particularly suitable for heat-sensitive compounds, such as polyphenols, vitamins, and essential oils, which may degrade under conventional drying methods. The freeze-drying process involves three primary stages:

‑ Freezing: The liquid vegetable extract is first rapidly frozen at temperatures below its freezing point (typically between −40 °C and −80 °C). This step ensures that the water within the extract forms ice crystals, which are essential for the subsequent sublimation phase. The freezing process also helps to retain the cellular structure of the plant material, which is crucial for preserving the quality of the extract.
‑ Primary Drying (Sublimation): During this phase, the frozen vegetable extract is placed under a vacuum, and heat is gently applied. This results in the sublimation of ice—where the ice transitions directly from a solid to a gas (vapor) without passing through the liquid phase. This stage removes the bulk of the water content (typically 90–95%) while keeping the bioactive compounds intact. The pressure and temperature are carefully controlled to prevent melting or damage to sensitive molecules.
‑ Secondary Drying (Desorption): After the majority of the water has been removed, the extract undergoes secondary drying to eliminate residual moisture (usually down to 1–5%). This is achieved by gradually increasing the temperature under vacuum conditions. The remaining water, which is bound to the molecules in the extract, is removed by desorption.

The result of freeze-drying is a stable, dry powder that retains most of the original properties of the vegetable extract, including its bioactive compounds, flavor, and color. The process is particularly advantageous for substances that are sensitive to heat, oxygen, and moisture.

To perform this study, the life cycle assessment (LCA) framework as defined by the ISO 14040 [34] and ISO 14044 standards [35] was followed. The LCA methodology consisted of four stages: Goal and Scope Definition, Life Cycle Inventory (LCI), Life Cycle Impact Assessment (LCIA), and Interpretation.

### 2.1. Goal and Scope Definition

The goal of this study was to evaluate and compare the environmental impacts associated with the production of tannin extracts from pine-derived forestry by-products (bark, cones, and pruning) through atomization and freeze-drying. Results were benchmarked against commercial tannins (mimosa, quebracho, and tara).

The functional unit was defined as 1000 kg of tannin equivalent, allowing for comparisons normalized by tannin concentration across all products. System boundaries included raw material acquisition, extraction, and processing stages, but excluded downstream use and end-of-life phases.

### 2.2. Life Cycle Inventory (LCI)

**Figure 1:**
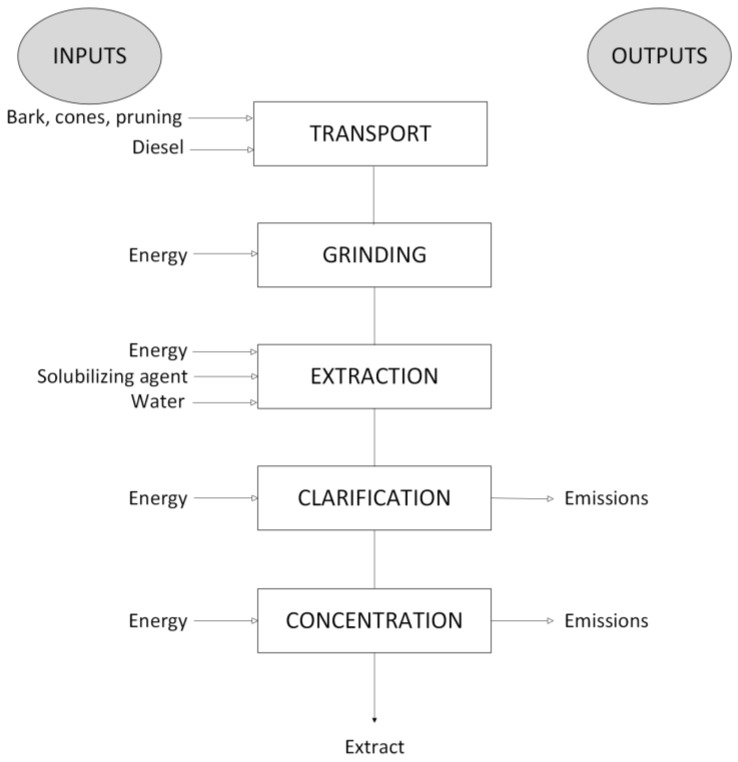
Flow chart of the process of obtaining an extract.

The inventory data were compiled from experimental trials and prior studies [29,30], supplemented by proxy data for substances not available in standard databases. A flow chart of the process of obtaining an extract can be seen in Figure 1.

Although, in general terms, the production of the different extracts involves the same unit processes, there are substantial differences between them depending on the raw material used, transportation, energy consumption, concentration method, and, most notably, the tannin content of each extract. For this reason, it is crucial to conduct a life cycle inventory that documents all input and output values for each extract. Beyond the intrinsic differences in each extract, this study seeks to evaluate the scalability challenges linked to the two concentration methods, atomization and freeze-drying, with a focus on identifying key environmental hotspots and assessing scalability limitations. Table 1, Table 2 and Table 3 present the input and output values for the extraction system of pine by-product extracts during the extraction phase and for the two concentration options: atomization and freeze-drying.

### 2.3. Life Cycle Impact Assessment (LCIA)

The environmental impacts were modeled using OpenLCA 1.11.0 with the Ecoinvent 3.8 database (January 2021). The EF3.0 midpoint method was applied to assess the impact categories that can be seen in Table 4.

All results were normalized to 1000 kg of tannin equivalent, with tannin concentrations for each material and process, as can be seen in Table 5. The tannin concentrations shown in Table 5 are the result of the average of three analyses. Through the use of these tannin concentrations for comparison, the evaluation can be conducted under equal conditions based on their industrial use.

### 2.4. Interpretation

The results were interpreted comparatively across products, focusing on relative contributions to impact categories. Energy use, particularly electricity and natural gas, was identified as the primary driver of impacts. Sensitivity analyses were conducted to evaluate the effect of increasing tannin concentrations in pine-derived products and the integration of renewable energy sources.

## 3. Results

This research investigated the production of polyphenolic extracts from three forestry by-products derived from pine trees: bark, cones, and pruning. The extraction process employed aqueous autoclaving, followed by concentration through atomization or freeze-drying to optimize tannin recovery. Comparative analyses with commercial tannin sources—mimosa, quebracho, and tara, as well as ground pine bark powder sieved to 2 mm—were performed to evaluate their environmental impacts as benchmarks. As mentioned before, this study uses EF3.0 midpoint categories for Life Cycle Impact Assessment (LCIA) and considers the tannin concentration in each product for a fair comparison. The results can be seen in Table 6.

**Figure 2:**
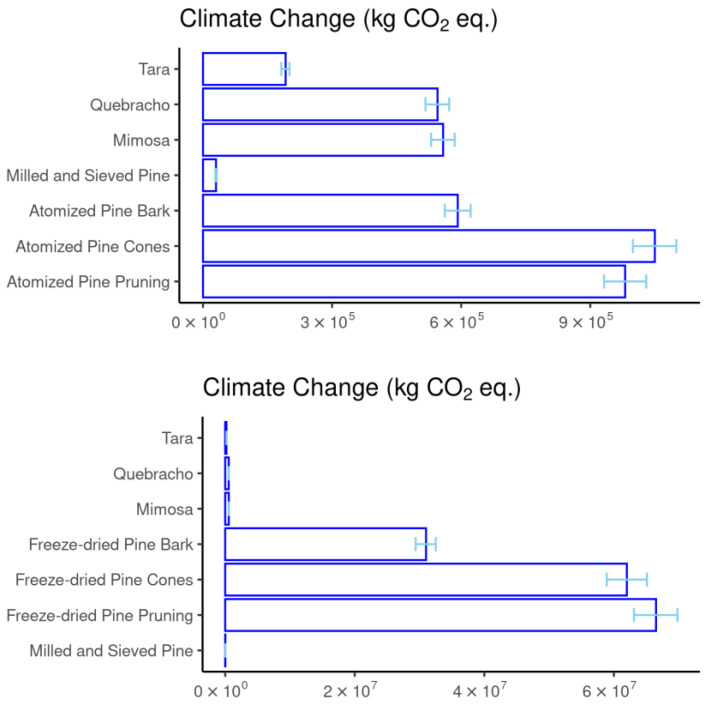
Climate change total results on an absolute scale (kg of CO_2_ equivalents).

Figure 2 presents the absolute results, such as climate change expressed in kg of CO_2_ equivalents, which is the impact category with the highest consensus regarding weighting factors in LCIAs. This category is also commonly used as an indicator of carbon footprint. 

The product with the best results is the chemically unmodified pine bark, that is, the ground pine bark powder sieved to 2 mm. Although unmodified pine bark powder causes much less environmental impact than the other extracts studied, it contains a large amount of insoluble substances, specifically around 60%. This makes its application in various sectors, such as leather, nutrition, or adhesives, more challenging. It can be observed that freeze-drying yields very high results, mainly because the values presented are from laboratory-scale equipment. The results of atomization are much more similar to commercial vegetable tannins, as they were obtained using semi-industrial pilot plant equipment (with a capacity to process 20 kg/h of product).

It is also noteworthy that ground and sieved pine has the lowest impact, due to its simpler process that does not require any chemicals and that consumes much less energy than the others. For the same reason, tara also falls in the lower range of the results. However, both extraction processes produce a product with a lower tannin concentration: 19,4% for the ground pine bark powder sieved to 2 mm and 35% for tara.

It is important to consider that absolute values in a life cycle analysis are subject to many parameters, making it more robust to study the comparative impact of products and processes, ensuring that the assumptions made affect the results equally.

**Figure 3:**
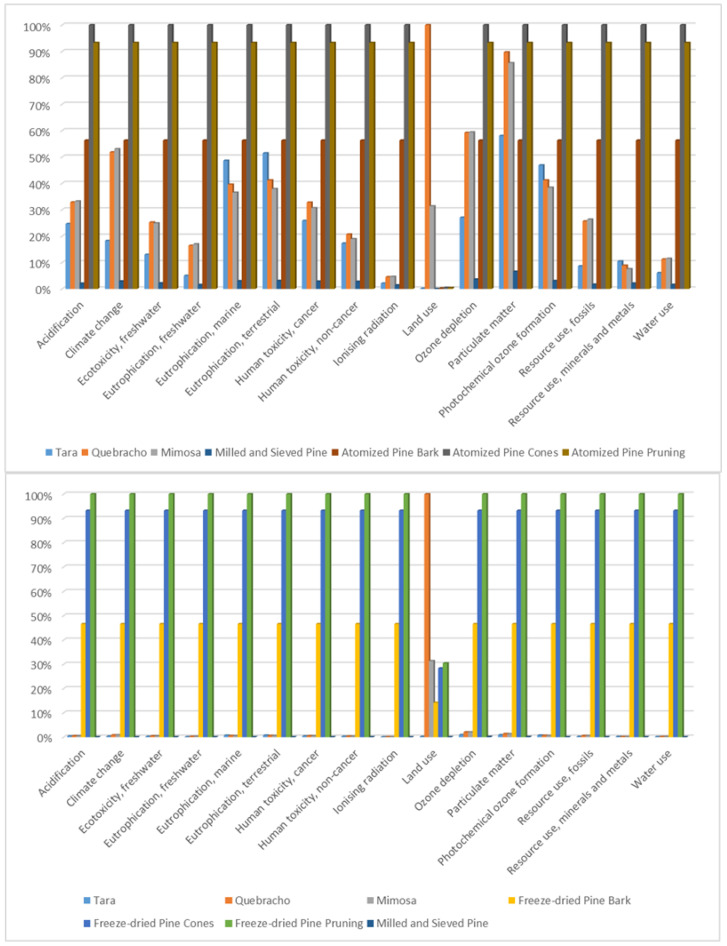
Comparative results of all EF3.0 categories on a relative scale.

To perform a comparative analysis, it is useful to compare values relatively so that the scale does not affect them between categories (in this case, the reference is taken as 100% of the highest value in each category, and the difference from the other products is observed). In Figure 3, this comparative analysis can be found, taking as a basis the atomized pine bark extract (at the top of the figure) and the freeze-dried pine bark extract (at the bottom of the figure).

In Figure 3, the dominant impact categories can be observed. To enable a quantitative analysis of the impact, Table 7 and Table 8 present the relative values for each process. The lowest values relative to the reference are shown in green, while significantly higher values relative to the reference are depicted in red. A gradient scale has been applied, where green represents the lowest values, yellow indicates the 50th percentile, and red denotes the highest values. The reference values selected are those of atomized pine bark and freeze-dried pine bark, respectively.

As shown in Table 7, the product with the best environmental performance is the chemically unmodified pine bark, specifically ground pine bark powder sieved to 2 mm. Additionally, it can be observed that pine needle extracts have 77.64% higher impacts compared to pine bark extracts, while extracts from pine pruning residues have 65.68% higher impacts. This highlights the concentration of the extract as a critical factor. Increasing the yield during the extraction and concentration processes could help reduce the environmental impact.

Commercial extracts such as tara, quebracho, and mimosa exhibit higher impacts in the category “Ecotoxicity, freshwater—organics”. This category refers to the environmental impact of toxic organic compounds when released into freshwater ecosystems. It evaluates how these compounds affect aquatic life, including fish, plants, and other aquatic organisms. The quebracho and mimosa extracts show 14% and 7% higher impacts, respectively, in the category “Human toxicity, cancer—organics”. This category evaluates the potential health risks posed by organic chemical substances that can cause cancer when released into the environment. The assessment involves several key factors:

‑ Environmental Fate Factor: This determines how the chemicals distribute and transform in the environment.
‑ Human Exposure Factor: This relates the amount of chemicals present in the environment to the level of human exposure.
‑ Human Toxicity Effect Factor: This indicates the potential toxic effects on humans per unit of chemical exposure.
‑ Damage Factor: This translates potential effects into actual health damage, often measured in disability-adjusted life years (DALYs).

In Table 7, a 5% increase in the “Ozone depletion” category can be seen for quebracho and mimosa. In the “Particulate matter” category, the impacts are 55% and 52% higher, respectively. However, the most significant differences are observed in the “Land use” category, where the impact of quebracho reaches 40,033.84% and that of mimosa is 12,555.96%. The land use impact category in a life cycle assessment (LCA) evaluates the environmental impacts associated with the occupation, transformation, and management of land for human purposes. This category considers both the long-term use of land (e.g., for agriculture) and changes in land use. Key aspects of this impact category include the following:

‑ Habitat Loss: Land use can lead to a reduction in natural habitats, affecting biodiversity and ecosystem services.
‑ Soil Degradation: Activities such as deforestation and intensive farming can degrade soil quality, reducing its fertility and increasing erosion.
‑ Water Regulation: Changes in land use can alter natural water cycles, affecting water availability and quality.
‑ Carbon Sequestration: Land use changes can impact the ability of ecosystems to sequester carbon, influencing greenhouse gas levels.

Table 8 presents the comparative results for the freeze-dried extracts, reaffirming that the product with the best environmental performance is the chemically unmodified pine bark. It is also confirmed that pine needle extracts and pruning residue extracts have 100% and 217.95% higher impacts, respectively, compared to pine bark extracts. This further emphasizes that the concentration of the extract is a critical factor in achieving better environmental impact results. Additionally, an increase in the “Land use” category is observed for quebracho and mimosa extracts. 

Table 9 shows the contributors to impacts for atomized pine bark extracts and freeze-dried pine bark extracts. The lowest values relative to the reference are shown in green, while significantly higher values relative to the reference are depicted in red. A gradient scale has been applied, where green represents the lowest values, yellow indicates the 50th percentile, and red denotes the highest values.

The primary contributors to the environmental impacts analyzed for the atomized and freeze-dried extracts are electricity and natural gas consumption in the case of atomization, and electricity consumption in the case of freeze-drying. Table 9 illustrate the main contributors for each impact category. As shown, the contribution of electricity to the atomized products generally exceeds 80%. For the freeze-dried products, electricity contributes over 99% in almost all categories, with the exceptions of “Ecotoxicity, freshwater—inorganics” and “Ozone depletion”.

## 4. Discussion

The present study includes both absolute (e.g., climate change in kg CO_2_ equivalents) and relative impact analyses of atomized and freeze-dried extracts from pine by-products. The results were compared against commercial tannins, with chemically unmodified pine bark powder serving as the reference product.

Although freeze-dried extracts are highly stable, lightweight, and easy to transport and store, and the preservation of bioactive compounds makes freeze-drying a preferred method in industries such as pharmaceuticals, nutraceuticals, and food production, freeze-drying is energy-intensive and time-consuming, which can make it costlier compared to other drying methods.

In the obtained results, it was observed that atomized pine bark performs better in terms of climate change impact due to its semi-industrial scalability and high tannin content (52.7%) compared to freeze-dried pine bark. Despite having a higher tannin content (62%), freeze-dried pine bark exhibits a greater environmental impact due to the energy-intensive laboratory-scale lyophilization process.

Electricity is the dominant contributor to environmental impacts across all categories, exceeding 80% for atomized products and 99% for freeze-dried ones. Natural gas also contributes significantly to the atomization process. These results align with previous studies conducted by Vauchel et al. [36] and Carlqvist et al. [1]. Both studies focus on polyphenol extraction under varied operational conditions, highlighting energy efficiency. As in our study, electricity dominates over all impacts, especially under laboratory conditions. The difference in energy impact between the lyophilization and atomization systems can be attributed to the fact that lyophilization was performed with laboratory-scale equipment, while atomization was conducted with semi-industrial-scale equipment. A previous study by Vega et al. [37] highlights the role of integrating techno-economic analysis with LCA. This is reflected in the current study’s scalability insights, showcasing the semi-industrial applicability of atomization.

Atomization is conducted using semi-industrial pilot plant equipment capable of processing up to 20 kg/h of product. This scalability highlights its feasibility for larger-scale production. While electricity is a significant contributor to environmental impacts, the energy demand for atomization is relatively lower compared to that for freeze-drying, and natural gas contributes significantly as a secondary energy source. Atomization achieves a tannin concentration of 52.7%, which is slightly lower than freeze-drying but sufficient for many industrial applications. Atomization’s established scalability makes it viable for industrial applications, enabling a streamlined process for integrating renewable energy sources to further reduce its environmental footprint. Freeze-drying remains constrained to laboratory-scale equipment, limiting its production capacity and making it unsuitable for large-scale applications without substantial investment in industrial-scale systems. Freeze-drying is highly energy-intensive, with electricity dominating over 99% of the environmental impact across all categories. The process involves prolonged energy use for freezing, sublimation, and secondary drying. Freeze-drying achieves a higher tannin concentration (62%) than atomization, offering superior product quality. However, this advantage is outweighed by its significant environmental and energy costs. The laboratory-scale nature of freeze-drying creates challenges in terms of economic and environmental sustainability. Scaling up would require addressing substantial energy requirements and optimizing process efficiency. While freeze-drying provides higher tannin concentrations, its scalability is hindered by its energy intensity and reliance on laboratory-scale processes. In contrast, atomization offers a more scalable and environmentally friendly alternative.

Apart from the high energy use of the two concentration systems studied, another parameter affecting the environmental impact is the tannin content in the final product obtained. These results also align with a previous study by Barjoveanu et al. [31], which emphasizes the importance of selecting biomass types with a high polyphenol content. This aligns with the recommendation to optimize tannin concentration in atomized pine bark.

**Figure 4:**
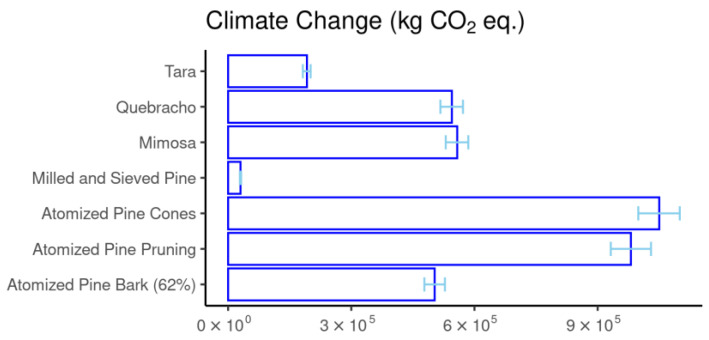
Total climate change results on an absolute scale (kg CO_2_ equivalents) according to the sensitivity analysis with increasing tannin concentrations.

In the present study, it was observed that lyophilization achieved the extraction of a 62% tannin content from pine bark. If the results obtained for the atomized samples (which reached 52.7%) were equivalent to those of the freeze-dried samples, the resulting impact would be lower than that of commercial atomized tannins (mimosa and quebracho), as shown in Figure 4. 

To enable a quantitative analysis of the impact, Table 9 presents the relative values for each process, showing a sensitivity analysis for increasing tannin concentrations of atomized pine bark extract. The lowest values relative to the reference are shown in green, while significantly higher values relative to the reference are depicted in red. A gradient scale has been applied, where green represents the lowest values, yellow indicates the 50th percentile, and red denotes the highest values.

The results in Table 10 indicate that an 85% reduction in the impact of pine bark leads to several favorable impact categories for atomized pine bark extract compared to mimosa and quebracho extracts. These categories include climate change (due to the fossil component), human ecotoxicity (subcategories: cancer—organics; non-cancer—inorganics), and ozone depletion. If the tannin content of pine bark were 62%, the climate change impact would already be lower than the reference values for quebracho and mimosa, which are also atomized extracts but require long-distance transport. Additionally, quebracho and mimosa extracts have a very high impact in the land use category.

The improvement proposals of this study are increasing the tannin content and integrating renewable energy. Enhancing the tannin concentration of atomized products would make them more competitive environmentally and economically. And shifting electricity sources to photovoltaic or wind power could significantly reduce the environmental footprint.

## 5. Conclusions

This study investigated the production of polyphenolic extracts from three forestry by-products derived from pine trees: bark, cones, and pruning. The extraction process employed aqueous autoclaving, followed by concentration through atomization or freeze-drying to optimize tannin recovery. Comparative analyses with commercial tannin sources—mimosa, quebracho, and tara, as well as ground pine bark powder sieved to 2 mm—were performed to evaluate their environmental impacts as benchmarks.

### 5.1. Environmental Impact of Extraction Methods

The results demonstrated that atomized pine bark performs better in terms of climate change impact due to its semi-industrial scalability and high tannin content (52.7%) compared to freeze-dried pine bark. Despite having a higher tannin content (62%), freeze-dried pine bark exhibits a greater environmental impact due to the energy-intensive laboratory-scale lyophilization process.

### 5.2. Contribution of Energy Sources

Electricity was identified as the dominant contributor to environmental impacts across all categories, exceeding 80% for atomized products and 99% for freeze-dried ones. Natural gas also contributed significantly to the atomization process.

### 5.3. Comparison with Commercial Tannins

This study also emphasized the importance of tannin concentration in the final product. A higher tannin content in atomized pine bark could potentially reduce its environmental impact, making it a competitive alternative to commercial tannins like mimosa and quebracho, which require long-distance transport and have high impacts in the land use category.

### 5.4. Recommendations for Improvement

The improvement proposals from this study include increasing the tannin content of atomized products and integrating renewable energy sources into the extraction process. These steps could further enhance the sustainability and environmental performance of pine-bark-derived tannins.

## Figures and Tables

**Table 1:** Log of inputs and outputs for the system used to obtain extracts from pine by-products.

Inputs		Outputs	
Extraction			
Flow	Amount	Flow	Amount
Pine Bark/Cone/Pruning (Functional Unit)	2 kg	Moist residues of pine by-products	3 kg
Water	10 kg	Liquid extract of pine bark/cones/pruning	9 kg
Solubilizing Agent	0.03 kg		
Electricity	5.607 kWh		

**Table 2:** Log of inputs and outputs for the system used to obtain atomized extracts from pine by-products.

Inputs		Outputs	
Atomization Process			
Pine Bark Extract	8380 kg–95 min	Atomized powdered pine bark extract (52.7% tannins)	438.6 g
Pine Pruning Extract	5140 kg–50 min	Atomized powdered pine pruning extract (39.4% tannins)	452.32 g
Pine Cone Extract	5100 kg–30 min	Atomized powdered pine cone extract (37.6% tannins)	913.42 g
Natural Gas	Bark: 10,056 kcal (0.80448 m^3^)	Distilled water condensates	7.9–8.5 kg
	Pruning: 6168 kcal (0.49344 m^3^)		
	Cone: 6120 kcal (0.4896 m^3^)		
Electricity	Bark: 1.676 kWh		
	Pruning: 1.028 kWh		
	Cone: 1.02 kWh		

**Table 3:** Log of inputs and outputs for the system used to obtain freeze-dried extracts from pine by-products.

Inputs		Outputs	
Freeze-Drying Process			
Pine Bark/Cone/Pruning Extract	318 g	Lyophilized powdered pine bark extract (62% tannins)	17.44 g
Electricity (Freezer)	(1.5 h) 0.678 kWh	Lyophilized powdered pine pruning extract (19.5% tannins)	
Electricity (Lyophilizer)	(45 h) 28.125 kWh	Lyophilized powdered pine cone extract (31% tannins)	
		Distilled water condensates	3163.56 g

**Table 4:** Descriptions of environmental impact categories.

Environmental Impact Category	Description	Reference Unit
Acidification	Decrease in soil and water pH as a result of emissions of NOx, SO_2_, NO_2_, NH_3_, HCl, HF, etc.	Mole H^+^ eq
Climate Change	Increase in Earth’s temperature due to the emission of greenhouse gases such as CO_2_, CH_4_, NO_x_, O_3_, etc. (fuel combustion, industrial emissions, etc.).	kg CO_2_ eq.
Depletion of Mineral Resources	For example: minerals like bauxite, limestone, iron, etc.	CTUe ^(1)^
Depletion of Fossil Resources	For example: fossil fuels like oil, natural gas, coal, etc.	kg P eq.
Eutrophication	Increase in inorganic nutrients (SO_4_^2−^ and NO_3_^−^) in water (excrement, fertilizers, etc.).	CTUh ^(2)^
Ecotoxicity	Pollutants that cause toxicity to ecosystems (plants and animals).	CTUh
Human Toxicity	Carcinogens: chemical compounds that cause cancer in humans.	CTUe
	Non-carcinogens: chemical compounds that cause diseases other than cancer.	kg U235 eq.
Respiratory Effects	Pollutants that cause respiratory diseases.	kg SOC
Use of Urban or Agricultural Land	Land use generates environmental impacts. Area of agricultural or urban land used and/or occupied due to industrial activity.	kg N eq.
Ozone Layer Depletion	The ozone layer acts as a filter for ultraviolet radiation reaching the Earth. Chlorofluorocarbon (CFC) compounds destroy this layer.	kg CFC^−11^ eq
Photochemical Oxidation (Smog)	Photochemical oxidants such as VOCs and NOx (from fuel combustion, industrial emissions, etc.) contribute to the formation of photochemical smog.	kg PM2.5 eq
Energy Consumed	Energy required throughout the life cycle of a product, including non-renewable energy from fossil fuels, nuclear sources, biomass, and renewable energy from solar, geothermal, wind, and hydro sources.	kg C_2_H_4_ eq.

^(1)^ CTUe: Ecosystem Toxicity Units (PAF × m^3^ × days/kg emitted); ^(2)^ CTUh: Human Toxicity Units (toxicity cases/kg emitted);

**Table 5:** Concentration of tannins for each extract.

Product	% Tannins	kg Equivalent to 1000 kg of Tannin
Commercial tara	35.0%	2857.14
Commercial quebracho	76.0%	1315.79
Commercial mimosa	67.0%	1492.54
Atomized powdered pine bark extract	52.7%	1897.53
Atomized powdered pine cone extract	37.6%	2659.57
Atomized powdered pine pruning extract	39.4%	2538.07
Ground and sifted pine	19.4%	5154.64
Lyophilized powdered pine bark extract	62.0%	1587.30
Lyophilized powdered pine cone extract	31.0%	3225.81
Lyophilized powdered pine pruning extract	28.9%	3460.21

**Table 6:** Results of the life cycle assessment.

Indicator	Tara	Quebracho	Mimosa	Freeze-Dried Pine Bark	Freeze-Dried Pine Cones	Freeze-Dried Pine Pruning	Milled and Sieved Pine	Atomized Pine Bark	Atomized Pine Cones	Atomized Pine Pruning	Atomized Pine Bark (62%)	Unit
**Acidification**	2.01 × 10^1^	2.67 × 10^1^	2.70 × 10^1^	2.69 × 10^3^	5.37 × 10^3^	5.76 × 10^3^	1.65 × 10^0^	4.58 × 10^1^	8.14 × 10^1^	7.59 × 10^1^	3.89 × 10^1^	mol H eq
**Climate change**	1.92 × 10^3^	5.45 × 10^3^	5.58 × 10^3^	3.10 × 10^5^	6.20 × 10^5^	6.65 × 10^5^	3.02 × 10^2^	5.92 × 10^3^	1.05 × 10^4^	9.81 × 10^3^	5.03 × 10^3^	kg CO₂ eq
**Climate change—Biogenic**	7.60 × 10^−1^	2.12 × 10^0^	2.19 × 10^0^	6.88 × 10^2^	1.38 × 10^3^	1.48 × 10^3^	2.95 × 10^−1^	1.14 × 10^1^	2.03 × 10^1^	1.89 × 10^1^	9.69 × 10^0^	kg CO₂ eq
**Climate change—Fossil**	1.92 × 10^3^	5.44 × 10^3^	5.57 × 10^3^	3.07 × 10^5^	6.13 × 10^5^	6.58 × 10^5^	3.01 × 10^2^	5.87 × 10^3^	1.04 × 10^4^	9.73 × 10^3^	4.99 × 10^3^	kg CO₂ eq
**Climate change—Land use and LU change**	1.21 × 10^0^	1.38 × 10^0^	1.36 × 10^0^	2.41 × 10^3^	4.81 × 10^3^	5.16 × 10^3^	8.44 × 10^−1^	3.76 × 10^1^	6.68 × 10^1^	6.23 × 10^1^	3.20 × 10^1^	kg CO₂ eq
**Ecotoxicity, freshwater**	2.47 × 10^4^	4.80 × 10^4^	4.74 × 10^4^	5.40 × 10^6^	1.08 × 10^7^	1.16 × 10^7^	4.10 × 10^3^	1.07 × 10^5^	1.90 × 10^5^	1.78 × 10^5^	9.11 × 10^4^	CTUe
**Ecotoxicity, freshwater—inorganics**	6.64 × 10^3^	7.50 × 10^3^	6.82 × 10^3^	2.11 × 10^5^	4.21 × 10^5^	4.52 × 10^5^	7.58 × 10^2^	1.33 × 10^4^	2.37 × 10^4^	2.21 × 10^4^	1.13 × 10^4^	CTUe
**Ecotoxicity, freshwater—metals**	1.68 × 10^4^	3.90 × 10^4^	3.92 × 10^4^	5.16 × 10^6^	1.03 × 10^7^	1.11 × 10^7^	3.17 × 10^3^	9.34 × 10^4^	1.66 × 10^5^	1.55 × 10^5^	7.94 × 10^4^	CTUe
**Ecotoxicity, freshwater—organics**	1.55 × 10^3^	1.80 × 10^3^	1.66 × 10^3^	4.20 × 10^4^	8.40 × 10^4^	9.01 × 10^4^	1.95 × 10^4^	7.51 × 10^2^	1.33 × 10^3^	1.25 × 10^3^	6.39 × 10^2^	CTUe
**Eutrophication, freshwater**	1.70 × 10^−1^	5.60 × 10^−1^	5.79 × 10^−1^	1.20 × 10^2^	2.40 × 10^2^	2.57 × 10^2^	5.26 × 10^−2^	1.92 × 10^0^	3.42 × 10^0^	3.19 × 10^0^	1.63 × 10^0^	kg P eq
**Eutrophication, marine**	6.06 × 10^0^	4.94 × 10^0^	4.55 × 10^0^	4.16 × 10^2^	8.33 × 10^2^	8.93 × 10^2^	3.68 × 10^−1^	7.02 × 10^0^	1.25 × 10^1^	1.16 × 10^1^	5.97 × 10^0^	kg N eq
**Eutrophication, terrestrial**	6.67 × 10^1^	5.34 × 10^1^	4.92 × 10^1^	4.33 × 10^3^	8.65 × 10^3^	9.28 × 10^3^	3.95 × 10^0^	7.30 × 10^1^	1.30 × 10^2^	1.21 × 10^2^	6.20 × 10^1^	mol N eq
**Human toxicity, cancer**	1.29 × 10^−6^	1.63 × 10^−6^	1.53 × 10^−6^	1.58 × 10^−4^	3.16 × 10^−4^	3.38 × 10^−4^	1.40 × 10^−7^	2.81 × 10^−6^	4.99 × 10^−6^	4.65 × 10^−6^	2.39 × 10^−6^	CTUh
**Human toxicity, cancer—metals**	5.90 × 10^−7^	6.35 × 10^−7^	5.93 × 10^−7^	1.15 × 10^−4^	2.30 × 10^−4^	2.47 × 10^−4^	8.39 × 10^−8^	1.93 × 10^−6^	3.43 × 10^−6^	3.20 × 10^−6^	1.64 × 10^−6^	CTUh
**Human toxicity, cancer—organics**	6.98 × 10^−7^	9.99 × 10^−7^	9.37 × 10^−7^	4.28 × 10^−5^	8.56 × 10^−5^	9.18 × 10^−5^	5.63 × 10^−8^	8.75 × 10^−7^	1.56 × 10^−6^	1.45 × 10^−6^	7.44 × 10^−7^	CTUh
**Human toxicity, non-cancer**	2.52 × 10^−5^	3.01 × 10^−5^	2.76 × 10^−5^	4.74 × 10^−3^	9.48 × 10^−3^	1.02 × 10^−2^	4.01 × 10^−6^	8.21 × 10^−5^	1.46 × 10^−4^	1.36 × 10^−4^	6.98 × 10^−5^	CTUh
**Human toxicity, non-cancer—inorganics**	5.03 × 10^−6^	5.96 × 10^−6^	5.48 × 10^−6^	2.44 × 10^−4^	4.87 × 10^−4^	5.23 × 10^−4^	6.39 × 10^−7^	6.25 × 10^−6^	1.11 × 10^−5^	1.03 × 10^−5^	5.31 × 10^−6^	CTUh
**Human toxicity, non-cancer—metals**	1.63 × 10^−5^	2.15 × 10^−5^	2.01 × 10^−5^	4.40 × 10^−3^	8.79 × 10^−3^	9.43 × 10^−3^	3.24 × 10^−6^	7.41 × 10^−5^	1.32 × 10^−4^	1.23 × 10^−4^	6.30 × 10^−5^	CTUh
**Human toxicity, non-cancer—organics**	3.85 × 10^−6^	2.34 × 10^−6^	1.64 × 10^−6^	1.16 × 10^−4^	2.32 × 10^−4^	2.49 × 10^−4^	1.38 × 10^−7^	2.87 × 10^−6^	5.09 × 10^−6^	4.75 × 10^−6^	2.44 × 10^−6^	CTUh
**Ionizing radiation**	1.24 × 10^2^	2.68 × 10^2^	2.75 × 10^2^	2.14 × 10^5^	4.29 × 10^5^	4.60 × 10^5^	8.30 × 10^1^	3.35 × 10^3^	5.95 × 10^3^	5.55 × 10^3^	2.85 × 10^3^	kBq U-235 eq
**Land use**	1.10 × 10^4^	6.67 × 10^6^	2.09 × 10^6^	9.46 × 10^5^	1.89 × 10^6^	2.03 × 10^6^	2.06 × 10^3^	1.67 × 10^4^	2.96 × 10^4^	2.76 × 10^4^	1.42 × 10^4^	Pt
**Ozone depletion**	3.92 × 10^−4^	8.58 × 10^−4^	8.61 × 10^−4^	1.98 × 10^−2^	3.97 × 10^−2^	4.26 × 10^−2^	5.23 × 10^−5^	8.16 × 10^−4^	1.45 × 10^−3^	1.35 × 10^−3^	6.94 × 10^−4^	kg CFC11 eq
**Particulate matter**	1.30 × 10^−4^	2.01 × 10^−4^	1.91 × 10^−4^	6.96 × 10^−3^	1.39 × 10^−2^	1.49 × 10^−2^	1.47 × 10^−5^	1.26 × 10^−4^	2.24 × 10^−4^	2.09 × 10^−4^	1.07 × 10^−4^	disease inc.
**Photochemical ozone formation**	1.80 × 10^1^	1.58 × 10^1^	1.47 × 10^1^	1.17 × 10^3^	2.33 × 10^3^	2.50 × 10^3^	1.16 × 10^0^	2.16 × 10^1^	3.83 × 10^1^	3.57 × 10^1^	1.83 × 10^1^	kg NMVOC eq
**Resource use, fossils**	2.71 × 10^4^	8.07 × 10^4^	8.29 × 10^4^	7.09 × 10^6^	1.42 × 10^7^	1.52 × 10^7^	5.28 × 10^3^	1.77 × 10^5^	3.15 × 10^5^	2.94 × 10^5^	1.51 × 10^5^	MJ
**Resource use, minerals and metals**	9.87 × 10^−3^	8.37 × 10^−3^	7.11 × 10^−3^	3.28 × 10^0^	6.56 × 10^0^	7.04 × 10^0^	1.95 × 10^−3^	5.32 × 10^−2^	9.44 × 10^−2^	8.81 × 10^−2^	4.52 × 10^−2^	kg Sb eq
**Water use**	1.86 × 10^2^	3.45 × 10^2^	3.53 × 10^2^	1.05 × 10^5^	2.11 × 10^5^	2.26 × 10^5^	4.80 × 10^1^	1.73 × 10^3^	3.07 × 10^3^	2.87 × 10^3^	1.47 × 10^3^	m³ depriv.

**Table 7:** Comparative results of all EF3.0 categories on a scale relative to the baseline process of atomized extracts.

Indicator	Tara	Quebracho	Mimosa	Milled and Sieved Pine	Atomized Pine Bark	Atomized Pine Cones	Atomized Pine Pruning	Atomized Pine Bark (62%)
Acidification	43.79%	58.35%	58.99%	3.61%	100.00%	177.64%	165.68%	85.00%
Climate change	32.40%	92.01%	94.20%	5.11%	100.00%	177.64%	165.68%	85.00%
Climate change—Biogenic	6.66%	18.60%	19.18%	2.59%	100.00%	177.64%	165.68%	85.00%
Climate change—Fossil	32.64%	92.72%	94.92%	5.13%	100.00%	177.64%	165.68%	85.00%
Climate change—Land use and LU change	3.21%	3.68%	3.62%	2.25%	100.00%	177.64%	165.68%	85.00%
Ecotoxicity, freshwater	23.07%	44.82%	44.24%	3.82%	100.00%	177.64%	165.68%	85.00%
Ecotoxicity, freshwater—inorganics	49.78%	56.22%	51.14%	5.68%	100.00%	177.64%	165.68%	85.00%
Ecotoxicity, freshwater—metals	17.95%	41.78%	41.96%	3.40%	100.00%	177.64%	165.68%	85.00%
Ecotoxicity, freshwater—organics	206.34%	239.87%	221.34%	25.91%	100.00%	177.64%	165.68%	85.00%
Eutrophication, freshwater	8.86%	29.14%	30.12%	2.73%	100.00%	177.64%	165.68%	85.00%
Eutrophication, marine	86.37%	70.30%	64.84%	5.24%	100.00%	177.64%	165.68%	85.00%
Eutrophication, terrestrial	91.40%	73.16%	67.35%	5.41%	100.00%	177.64%	165.68%	85.00%
Human toxicity, cancer	45.89%	58.19%	54.46%	4.99%	100.00%	177.64%	165.68%	85.00%
Human toxicity, cancer—metals	30.54%	32.85%	30.66%	4.34%	100.00%	177.64%	165.68%	85.00%
Human toxicity, cancer—organics	79.79%	114.14%	107.02%	6.44%	100.00%	177.64%	165.68%	85.00%
Human toxicity, non-cancer	30.66%	36.69%	33.57%	4.89%	100.00%	177.64%	165.68%	85.00%
Human toxicity, non-cancer—inorganics	80.59%	95.42%	87.79%	10.23%	100.00%	177.64%	165.68%	85.00%
Human toxicity, non-cancer—metals	22.00%	29.09%	27.17%	4.38%	100.00%	177.64%	165.68%	85.00%
Human toxicity, non-cancer—organics	134.22%	81.58%	57.34%	4.80%	100.00%	177.64%	165.68%	85.00%
Ionizing radiation	3.69%	8.00%	8.21%	2.48%	100.00%	177.64%	165.68%	85.00%
Land use	66.10%	40,033.84%	12,555.96%	12.34%	100.00%	177.64%	165.68%	85.00%
Ozone depletion	48.00%	105.15%	105.49%	6.40%	100.00%	177.64%	165.68%	85.00%
Particulate matter	103.10%	159.37%	152.10%	11.64%	100.00%	177.64%	165.68%	85.00%
Photochemical ozone formation	83.25%	73.17%	68.21%	5.39%	100.00%	177.64%	165.68%	85.00%
Resource use, fossils	15.30%	45.55%	46.77%	2.98%	100.00%	177.64%	165.68%	85.00%
Resource use, minerals and metals	18.57%	15.75%	13.38%	3.68%	100.00%	177.64%	165.68%	85.00%
Water use	10.74%	19.91%	20.38%	2.78%	100.00%	177.64%	165.68%	85.00%

**Table 8:** Comparative results of all EF3.0 categories on a scale relative to the baseline process of freeze-dried extracts.

Indicator	Tara	Quebracho	Mimosa	Freeze-Dried Pine Bark	Freeze-Dried Pine Cones	Freeze-Dried Pine Pruning	Milled and Sieved Pine
Acidification	0.75%	1.00%	1.01%	100.00%	200.00%	214.53%	0.06%
Climate change	0.62%	1.76%	1.80%	100.00%	200.00%	214.53%	0.10%
Climate change—Biogenic	0.11%	0.31%	0.32%	100.00%	200.00%	214.53%	0.04%
Climate change—Fossil	0.62%	1.77%	1.82%	100.00%	200.00%	214.53%	0.10%
Climate change—Land use and LU change	0.05%	0.06%	0.06%	100.00%	200.00%	214.53%	0.04%
Ecotoxicity, freshwater	0.46%	0.89%	0.88%	100.00%	200.00%	214.53%	0.08%
Ecotoxicity, freshwater—inorganics	3.15%	3.56%	3.24%	100.00%	200.00%	214.53%	0.36%
Ecotoxicity, freshwater—metals	0.33%	0.76%	0.76%	100.00%	200.00%	214.53%	0.06%
Ecotoxicity, freshwater—organics	3.69%	4.29%	3.96%	100.00%	200.00%	214.53%	0.46%
Eutrophication, freshwater	0.14%	0.47%	0.48%	100.00%	200.00%	214.53%	0.04%
Eutrophication, marine	1.46%	1.19%	1.09%	100.00%	200.00%	214.53%	0.09%
Eutrophication, terrestrial	1.54%	1.23%	1.14%	100.00%	200.00%	214.53%	0.09%
Human toxicity, cancer	0.82%	1.04%	0.97%	100.00%	200.00%	214.53%	0.09%
Human toxicity, cancer—metals	0.51%	0.55%	0.52%	100.00%	200.00%	214.53%	0.07%
Human toxicity, cancer—organics	1.63%	2.34%	2.19%	100.00%	200.00%	214.53%	0.13%
Human toxicity, non-cancer	0.53%	0.64%	0.58%	100.00%	200.00%	214.53%	0.08%
Human toxicity, non-cancer—inorganics	2.07%	2.45%	2.25%	100.00%	200.00%	214.53%	0.26%
Human toxicity, non-cancer—metals	0.37%	0.49%	0.46%	100.00%	200.00%	214.53%	0.07%
Human toxicity, non-cancer—organics	3.32%	2.02%	1.42%	100.00%	200.00%	214.53%	0.12%
Ionizing radiation	0.06%	0.12%	0.13%	100.00%	200.00%	214.53%	0.04%
Land use	1.16%	705.28%	221.20%	100.00%	200.00%	214.53%	0.22%
Ozone depletion	1.98%	4.33%	4.34%	100.00%	200.00%	214.53%	0.26%
Particulate matter	1.87%	2.88%	2.75%	100.00%	200.00%	214.53%	0.21%
Photochemical ozone formation	1.54%	1.35%	1.26%	100.00%	200.00%	214.53%	0.10%
Resource use, fossils	0.38%	1.14%	1.17%	100.00%	200.00%	214.53%	0.07%
Resource use, minerals and metals	0.30%	0.26%	0.22%	100.00%	200.00%	214.53%	0.06%
Water use	0.18%	0.33%	0.33%	100.00%	200.00%	214.53%	0.05%

**Table 9:** Summary table of contributors to impacts for atomized pine bark extracts (left) and freeze-dried pine bark extracts (right).

Impact Category (Atomized Bark)	Reference Unit	Electricity	Natural Gas	Sodium Hydroxide		Impact Category (Freeze-Dried Pine Bark)	Reference Unit	Electricity	Sodium Hydroxide
Acidification	mol H+ eq	90.8%	8.3%	0.9%		Acidification	mol H+ eq	99.7%	0.3%
Climate change	kg CO_2_ eq	80.9%	17.8%	1.3%		Climate change	kg CO_2_ eq	99.6%	0.4%
Climate change—Biogenic	kg CO_2_ eq	93.1%	4.9%	2.0%		Climate change—Biogenic	kg CO_2_ eq	99.4%	0.6%
Climate change—Fossil	kg CO_2_ eq	80.8%	18.0%	1.3%		Climate change—Fossil	kg CO_2_ eq	99.6%	0.4%
Climate change—Land use and LU change	kg CO_2_ eq	99.2%	0.4%	0.4%		Climate change—Land use and LU change	kg CO_2_ eq	99.9%	0.1%
Ecotoxicity, freshwater	CTUe	77.7%	20.6%	1.7%		Ecotoxicity, freshwater	CTUe	99.4%	0.6%
Ecotoxicity, freshwater—inorganics	CTUe	24.0%	74.0%	2.1%		Ecotoxicity, freshwater—inorganics	CTUe	97.8%	2.2%
Ecotoxicity, freshwater—metals	CTUe	85.3%	13.1%	1.7%		Ecotoxicity, freshwater—metals	CTUe	99.5%	0.5%
Ecotoxicity, freshwater—organics	CTUe	86.4%	12.4%	1.2%		Ecotoxicity, freshwater—organics	CTUe	99.6%	0.4%
Eutrophication, freshwater	kg P eq	96.3%	1.9%	1.9%		Eutrophication, freshwater	kg P eq	99.5%	0.5%
Eutrophication, marine	kg N eq	91.7%	7.0%	1.3%		Eutrophication, marine	kg N eq	99.6%	0.4%
Eutrophication, terrestrial	mol N eq	91.7%	7.1%	1.2%		Eutrophication, terrestrial	mol N eq	99.7%	0.3%
Human toxicity, cancer	CTUh	86.7%	11.6%	1.7%		Human toxicity, cancer	CTUh	99.5%	0.5%
Human toxicity, cancer—metals	CTUh	91.8%	6.4%	1.8%		Human toxicity, cancer—metals	CTUh	99.5%	0.5%
Human toxicity, cancer—organics	CTUh	75.5%	23.1%	1.4%		Human toxicity, cancer—organics	CTUh	99.5%	0.5%
Human toxicity, non-cancer	CTUh	89.1%	9.0%	1.9%		Human toxicity, non-cancer	CTUh	99.4%	0.6%
Human toxicity, non-cancer—inorganics	CTUh	59.6%	36.8%	3.6%		Human toxicity, non-cancer—inorganics	CTUh	98.4%	1.6%
Human toxicity, non-cancer—metals	CTUh	91.6%	6.6%	1.8%		Human toxicity, non-cancer—metals	CTUh	99.5%	0.5%
Human toxicity, non-cancer—organics	CTUh	62.4%	36.2%	1.3%		Human toxicity, non-cancer—organics	CTUh	99.4%	0.6%
Ionizing radiation	kBq U-235 eq	99.3%	0.4%	0.3%		Ionizing radiation	kBq U-235 eq	99.9%	0.1%
Land use	Pt	87.8%	11.2%	1.0%		Land use	Pt	99.7%	0.3%
Ozone depletion	kg CFC11 eq	36.2%	58.2%	5.6%		Ozone depletion	kg CFC11 eq	96.1%	3.9%
Particulate matter	disease inc.	85.0%	12.3%	2.7%		Particulate matter	disease inc.	99.2%	0.8%
Photochemical ozone formation	kg NMVOC eq	83.7%	15.2%	1.1%		Photochemical ozone formation	kg NMVOC eq	99.7%	0.3%
Resource use, fossils	MJ	61.9%	37.5%	0.5%		Resource use, fossils	MJ	99.8%	0.2%
Resource use, minerals and metals	kg Sb eq	95.2%	2.8%	2.0%		Resource use, minerals and metals	kg Sb eq	99.5%	0.5%
Water use	m3 depriv.	93.3%	1.9%	4.8%		Water use	m3 depriv.	98.7%	1.3%

**Table 10:** Comparative results for all EF3.0 categories on a scale relative to the baseline process, including sensitivity analysis.

Indicator	Tara	Quebracho	Mimosa	Milled and Sieved Pine	Atomized Pine Bark	Atomized Pine Cones	Atomized Pine Pruning	Atomized Pine Bark (62%)
Acidification	51.52%	68.65%	69.40%	4.25%	117.65%	208.99%	194.91%	100.00%
Climate change	38.12%	108.25%	110.82%	6.01%	117.65%	208.99%	194.91%	100.00%
Climate change—Biogenic	7.84%	21.88%	22.56%	3.05%	117.65%	208.99%	194.91%	100.00%
Climate change—Fossil	38.40%	109.08%	111.67%	6.04%	117.65%	208.99%	194.91%	100.00%
Climate change—Land use and LU change	3.77%	4.33%	4.25%	2.64%	117.65%	208.99%	194.91%	100.00%
Ecotoxicity, freshwater	27.14%	52.73%	52.04%	4.50%	117.65%	208.99%	194.91%	100.00%
Ecotoxicity, freshwater—inorganics	58.56%	66.14%	60.17%	6.69%	117.65%	208.99%	194.91%	100.00%
Ecotoxicity, freshwater—metals	21.12%	49.15%	49.37%	4.00%	117.65%	208.99%	194.91%	100.00%
Ecotoxicity, freshwater—organics	242.75%	282.20%	260.40%	30.48%	117.65%	208.99%	194.91%	100.00%
Eutrophication, freshwater	10.43%	34.28%	35.43%	3.22%	117.65%	208.99%	194.91%	100.00%
Eutrophication, marine	101.61%	82.70%	76.29%	6.16%	117.65%	208.99%	194.91%	100.00%
Eutrophication, terrestrial	107.53%	86.07%	79.23%	6.36%	117.65%	208.99%	194.91%	100.00%
Human toxicity, cancer	53.99%	68.46%	64.07%	5.87%	117.65%	208.99%	194.91%	100.00%
Human toxicity, cancer—metals	35.93%	38.65%	36.08%	5.11%	117.65%	208.99%	194.91%	100.00%
Human toxicity, cancer—organics	93.88%	134.28%	125.90%	7.57%	117.65%	208.99%	194.91%	100.00%
Human toxicity, non-cancer	36.07%	43.17%	39.49%	5.75%	117.65%	208.99%	194.91%	100.00%
Human toxicity, non-cancer—inorganics	94.81%	112.26%	103.28%	12.03%	117.65%	208.99%	194.91%	100.00%
Human toxicity, non-cancer—metals	25.89%	34.22%	31.97%	5.15%	117.65%	208.99%	194.91%	100.00%
Human toxicity, non-cancer—organics	157.91%	95.98%	67.46%	5.64%	117.65%	208.99%	194.91%	100.00%
Ionizing radiation	4.35%	9.41%	9.66%	2.91%	117.65%	208.99%	194.91%	100.00%
Land use	77.77%	47,098.52%	14,771.69%	14.52%	117.65%	208.99%	194.91%	100.00%
Ozone depletion	56.47%	123.70%	124.10%	7.54%	117.65%	208.99%	194.91%	100.00%
Particulate matter	121.29%	187.50%	178.94%	13.69%	117.65%	208.99%	194.91%	100.00%
Photochemical ozone formation	97.94%	86.09%	80.25%	6.34%	117.65%	208.99%	194.91%	100.00%
Resource use, fossils	18.00%	53.59%	55.02%	3.50%	117.65%	208.99%	194.91%	100.00%
Resource use, minerals and metals	21.85%	18.53%	15.74%	4.32%	117.65%	208.99%	194.91%	100.00%
Water use	12.64%	23.42%	23.97%	3.26%	117.65%	208.99%	194.91%	100.00%

## Data Availability

The original contributions presented in this study are included in the article. Further inquiries can be directed to the corresponding author.
